# Exploration of the advantages of minimally invasive surgery for clinical T4 colorectal cancer compared with open surgery: A matched-pair analysis

**DOI:** 10.1097/MD.0000000000029869

**Published:** 2022-08-12

**Authors:** Ken Imaizumi, Shigenori Homma, Yoichi Miyaoka, Hiroki Matsui, Nobuki Ichikawa, Tadashi Yoshida, Norihiko Takahashi, Akinobu Taketomi

**Affiliations:** a Department of Gastroenterological Surgery I, Graduate School of Medicine, Hokkaido University, Sapporo, Hokkaido, Japan.

**Keywords:** chemotherapy, clinical T4, colorectal cancer, minimally invasive surgery, primary tumor resection

## Abstract

The indications of minimally invasive surgery (MIS) for T4 colorectal cancer are controversial because the advantages of MIS are unclear. Therefore, we compared overall survival (OS) and recurrence-free survival (RFS) as the primary endpoint, and short-term outcome, alteration in perioperative laboratory data, and the interval of postoperative chemotherapy from operation as secondary endpoints, between MIS and open surgery (OPEN) using a matched-pair analysis. We explored the advantages of MIS for T4 colorectal cancer.

In this retrospective single-institution study, we included 125 patients with clinical T4 colorectal cancer who underwent curative-intent surgery of the primary tumor between October 2010 and September 2019. Conversion cases were excluded. MIS patients were matched to OPEN patients (ratio of 1:2) according to tumor location, clinical T stage, and preoperative treatment.

We identified 25 and 50 patients who underwent OPEN and MIS, respectively, including 31 with distant metastasis. Both groups had similar background characteristics. The rate of major morbidities (Clavien-Dindo grade *>* III) was comparable between the 2 groups (*P* = .597), and there was no mortality in either group. MIS tended to result in shorter postoperative hospitalization than OPEN (*P* = .073). Perioperative alterations in laboratory data revealed that MIS suppressed surgical invasiveness better compared to OPEN. Postoperative chemotherapy, especially for patients with distant metastasis who underwent primary tumor resection, tended to be started earlier in the MIS group than in the OPEN group (*P* = .075). OS and RFS were comparable between the 2 groups (*P* = .996 and .870, respectively). In the multivariate analyses, MIS was not a significant prognostic factor for poor OS and RFS.

MIS was surgically safe and showed similar oncological outcomes to OPEN—with the potential of reduced invasiveness and enhanced recovery from surgery. Therefore, patients undergoing MIS might receive subsequent postoperative treatments earlier.

## 1. Introduction

Minimally invasive surgery (MIS) is commonly performed for the resection of colorectal cancer (CRC), as it has short-term benefits and is oncologically safe.^[1–9]^ However, the safety and feasibility of MIS have not been established for advanced CRC, especially T4 CRC. For T4 CRC, the surgical procedure is extremely difficult because en bloc resection of the adjacent organs or structures is generally required for preserving the surgical resection margin. However, open multivisceral resection (MVR) for T4 CRC has a high rate of postoperative morbidity and a high risk of microscopically positive surgical margins.^[10–12]^ Moreover, major randomized trials comparing laparoscopy and open surgery (OPEN) excluded patients with T4 tumors, perforated tumors, and acute bowel obstruction.^[1–7]^ Although the JCOG 0404 trial showed that pathological T4 colon cancer was associated with poor prognostic factors,^[9]^ some retrospective studies showed that MIS was safe and feasible for T4 CRC, with good oncologic outcomes.^[12–21]^ However, these retrospective studies included a relatively small number of patients or were 1-arm studies in which researchers did not compare MIS with OPEN.^[12–17,21]^

After surgery for T4 CRC, patients without distant metastasis might receive adjuvant chemotherapy. Moreover, patients with T4 CRC frequently develop synchronous distant metastases^[14–17]^ and require additional treatment after primary tumor resection. Accordingly, the National Comprehensive Cancer Network Guidelines do not recommend primary tumor resection for CRC with distant metastasis. However, most patients with T4 CRC develop severe symptoms associated with the primary tumor and might require primary tumor resection. Hence, MIS, which is less invasive and results in quick postoperative recovery, can be performed as the subsequent treatment, and may result in an improved prognosis.

Therefore, in the current study, we aimed to evaluate the short-term and long-term outcomes between MIS and OPEN by using a matched-pair method, and to explore the advantages of MIS for T4 CRC during the perioperative course.

## 2. Methods

### 2.1. Study design and patients

This retrospective single-institution study was conducted in accordance with the Helsinki Declaration and was approved by the local institutional review board (Sapporo, Japan; No. 019-0320). The requirement for acquisition of informed consent from patients was waived owing to the retrospective nature of this study.

**Figure 1.**
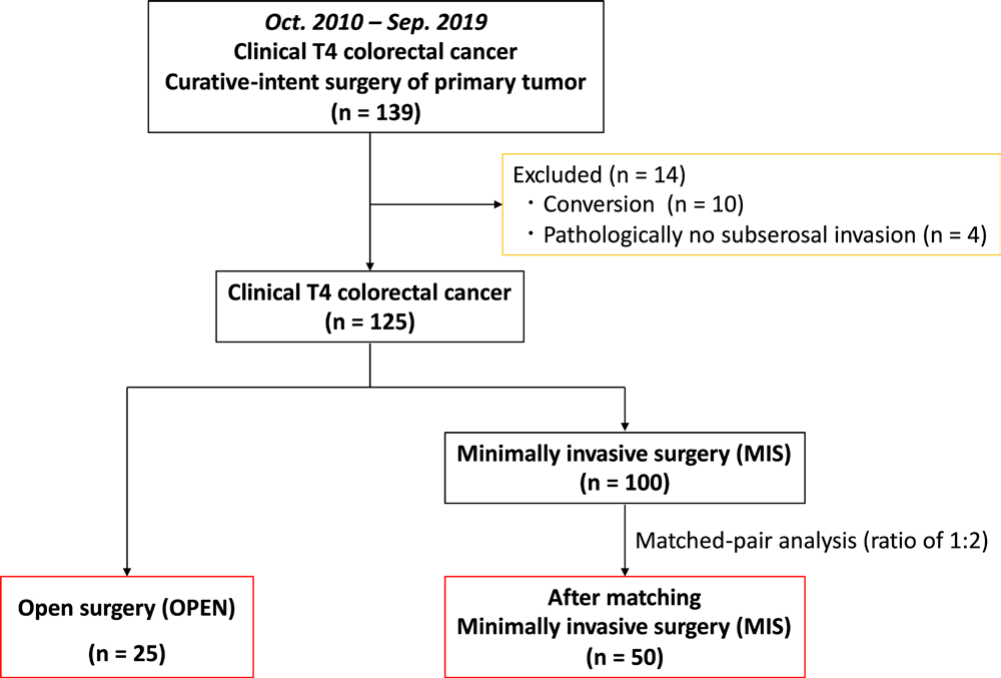
Patient flow and selection algorithm. MIS = minimally invasive surgery, OPEN = open surgery.

In our department, 696 consecutive patients underwent tumor resection for CRC between October 2010 and September 2019; we included 139 patients who were preoperatively diagnosed with clinical T4 CRC by routine examination, colonoscopy, and enhanced computed tomography. In cases of preoperative treatments, those with clinical T4 CRC based on examinations before treatments were included. Patients with distant metastasis who underwent resection of the primary tumor were also included. All patients underwent curative-intent surgery of the primary tumor. Patients with palliative surgery were not included. Because we compared complete MIS with OPEN in the current retrospective exploratory study to determine the advantages of MIS, 10 patients with conversion from MIS to OPEN were excluded due to the following reasons: 5 cases of a huge tumor, 2 cases of massive adhesion, 2 cases of insufficient visualization by adjacent organs invasion, and 1 case of bleeding. Four patients with pathological findings of no subserosal invasion (less than T3) were also excluded. Finally, we included 125 patients with clinical T4 CRC in this study. Twenty-five patients underwent OPEN and 100 underwent MIS. Among the 100 patients who underwent MIS, 91 underwent conventional laparoscopic surgery, 6 underwent reduced port surgery, and 3 underwent robotic-assisted surgery. To reduce the imbalance and selection bias between the OPEN and MIS groups, OPEN cases were compared to 50 MIS cases using a matched-pair analysis (ratio of 1:2) (Fig. 1). The matching parameters were the following 3 independent confounders: tumor location, clinical T stage, and preoperative treatment.

Clinical data were retrieved from the medical records. The patient background, pathological findings for the primary tumor, and clinical outcomes were reviewed. Clinical data and pathological information were prospectively recorded in a single database.

Considering the tumor location, the right colon was defined as the colon from the cecum to the transverse colon near the splenic flexure, and the left colon was defined as the colon from the descending to the rectosigmoid colon. The tumor stage was determined according to the eighth American Joint Committee on Cancer TNM edition. According to these guidelines, T4 tumors were subcategorized into T4a and T4b tumors. T4a tumors invaded the visceral peritoneum, including gross perforation of the bowel by the tumor and continuous invasion of the tumor through areas of inflammation to the surface of the visceral peritoneum. T4b tumors directly invaded or adhered to other adjacent organs or structures. Histological grades were classified as low (well or moderately differentiated adenocarcinoma) or high (poorly differentiated or mucinous adenocarcinoma or signet-ring cell carcinoma or neuroendocrine carcinoma).

### 2.2. Study endpoints

The primary endpoint was a difference in overall survival (OS) and recurrence-free survival (RFS) between MIS and OPEN for clinical T4 CRC. The secondary endpoints were differences in short-term outcome and perioperative laboratory data between MIS and OPEN. We also evaluated the interval of postoperative chemotherapy from operation in MIS and OPEN.

### 2.3. Surgical procedures

Conventional laparoscopic surgery was performed using a 5-port technique, while reduced port surgery was performed using a multichannel port and an additional port. For abdominoperineal resection, a multichannel port was inserted in the right or left lower quadrant at the planned colostomy site and an additional port in the umbilicus. For anterior resection, a multichannel port was inserted in the umbilicus and an additional port in the right lower quadrant. All these procedures have been reported previously.^[22,23]^ Robotic-assisted surgery was performed using a 6-port technique. The operative principles for dual-port laparoscopic and robotic-assisted surgeries were the same as those used for conventional laparoscopic surgery. The procedures were categorized as right-sided colectomy, left-sided colectomy, rectal resection, and total colectomy. Right-sided colectomy included ileocecal resection, right hemicolectomy, and extended right hemicolectomy for tumors located from the cecum to the transverse colon. Left-sided colectomy was defined as the resection of tumors from the splenic flexure to the sigmoid colon. MVR was defined as en bloc resection of any organ or structure to which the primary tumor adhered. Reconstruction after MVR included digestive or urinary tract reconstruction. In our department, the surgical indications for T4 CRC have been expanded gradually. Initially, MIS was introduced for T4a tumors treated without MVR, then for cases requiring MVR without reconstruction, and finally, for cases requiring MVR with reconstruction. Currently, the indications for the conventional laparoscopic approach include all T4 cases treated with or without reconstruction. However, reduced port and robotic approaches are not introduced for cases that are treated with reconstruction. Postoperative morbidity and mortality were defined as events occurring during the hospital stay or within 30 days of surgery. Postoperative complications were assessed by using the Clavien-Dindo classification.^[24]^ Major morbidities were defined as grade *>*III morbidities per the Clavien-Dindo classification.

### 2.4. Laboratory tests

Venous blood samples were collected for routine tests within 2 weeks prior to surgery, and on the morning of postoperative day (POD) 1, 3, and 7. White blood cell, neutrophil, and lymphocyte counts, as well as C-reactive protein (CRP) and serum total protein (TP) levels, were routinely measured at a central laboratory. The data were not included in the analysis if the patients did not have available data for any one of the 4 above-mentioned time points.

### 2.5. Postoperative chemotherapy and follow-up

After surgery, oncologists recommended adjuvant chemotherapy for all patients with pathological stage (pStage) III tumors, patients with pStage II tumors with high-risk factors, or patients with pStage IV tumors treated with curative resection for the primary tumor and distant metastasis. In addition, they recommended systemic chemotherapy for pStage II and III tumors with macroscopic residual disease of the primary tumor as well as for patients with pStage IV tumors treated with primary tumor resection, unless there were contraindications owing to a patient’s performance status. Oxaliplatin-based regimens were the most commonly used adjuvant chemotherapy regimens. Systemic chemotherapy regimens varied, and were administered at the oncologists’ discretion. All patients were followed up regularly after surgery. The follow-up time was calculated as the time interval from the date of the operation until the last follow-up date or death.

### 2.6. Statistical analysis

Categorical variables were compared using the Fisher exact test. Continuous variables were assessed using the 2-tailed Student *t* test and nonparametric Mann–Whitney *U* test. OS and RFS were estimated using the Kaplan–Meier method, and the differences between the survival curves were evaluated using a log-rank test. Prognostic factors for OS were analyzed using a multivariate analysis with a Cox proportional hazards regression model. *P*-values <.05 were considered statistically significant. All statistical analyses were performed with EZR (Saitama Medical Centre, Jichi Medical University, Saitama, Japan, version 1.50), which is a graphical user interface for *R* (The *R* Foundation for Statistical Computing, Vienna, Austria, version 3.6.3). More precisely, it is a modified version of *R* Commander (version 2.6-2) that is designed to add statistical functions frequently used in biostatistics.^[25]^

## 3. Results

### 3.1. Patient characteristics

The characteristics of the included patients are shown in Table 1. Before matching, the rates of previous abdominal surgery, clinical T factor, and preoperative treatment were significantly different between the OPEN and MIS groups. After matching, patients’ characteristics were similar between the OPEN and MIS groups. There were more cases with clinical T4b CRC in the OPEN group (56%) than in the MIS group (42%), but the difference was not significant (*P* = .328). Preoperative treatments, such as chemotherapy and chemoradiotherapy, were performed for 9 (36%) and 12 (24%) patients in the OPEN and MIS groups, respectively, but the difference was not significant (*P* = .289). Among patients with rectal cancer, 3 in the OPEN group received chemotherapy, whereas 3 received chemotherapy and 3 received chemoradiotherapy in the MIS group.

**Table 1:** Comparison of patient characteristics between OPEN and MIS before or after matching.

Characteristics			Before matching		After matching	
		OPEN (n = 25), n (%)	MIS (n = 100), n (%)	*P* value	MIS (n = 50), n (%)	*P* value
Age (y)*		64 (20–84)	67.5 (33–89)	.216	66.5 (41–87)	.202
Sex	Male	15 (60%)	50 (50.0%)	.503	25 (50%)	.468
	Female	10 (40%)	50 (50.0%)		25 (50%)	
Body mass index (kg/m^2^)*		21.8 (16.6–27.8)	21.5 (9.7–40.6)	.993	21.7 (15.5–40.6)	.888
ASA physical status	1	2 (8%)	24 (24%)	.084	10 (20%)	.274
	2	18 (72%)	67 (67%)		35 (70%)	
	3	5 (20%)	9 (9%)		5 (10%)	
Previous open abdominal surgery†	Total	15 (60%)	29 (29%)	.005	18 (36%)	.083
	Appendectomy	2 (8%)	14 (14%)	.037	7 (14%)	.169
	Gynecological surgery	5 (20%)	10 (10%)		8 (16%)	
	Gastrointestinal surgery	5 (20%)	2 (2%)		2 (4%)	
	Cholecystectomy	0 (0%)	1 (1%)		0 (0%)	
	Aortic surgery	1 (4%)	0 (0%)		0 (0%)	
	Unknown	2 (8%)	3 (3%)		1 (2%)	
Tumor location	Right colon	5 (20%)	40 (40%)	.146	13 (26%)	.900
	Left colon	13 (52%)	41 (41%)		25 (50%)	
	Rectum	7 (28%)	19 (19%)		12 (24%)	
Clinical T factor	T4a	11 (44%)	77 (77%)	.003	29 (58%)	.328
	T4b	14 (56%)	23 (23%)		21 (42%)	
Nodal involvement		16 (64%)	73 (73%)	.459	34 (68%)	.797
Distant metastasis		10 (40%)	44 (44%)	.823	21 (42%)	1.000
Preoperative treatment (chemotherapy or chemoradiotherapy)		9 (36%)	12 (12%)	.013	12 (24%)	.289

### 3.1. Surgical outcomes and perioperative alterations in laboratory data

The surgical data are shown in Table 2. The surgical procedure was comparable between the OPEN and MIS groups. Total colectomy was performed for only 1 patient with transverse colon cancer and a peritoneal nodule at the sigmoid colon. MVR for invasive and adherent tumors and lymph node harvest were also comparable between the 2 groups. However, the OPEN group underwent more MVRs with reconstruction than the MIS group (e.g., resection of total bladder with urinary reconstruction). Accordingly, the surgical time was significantly longer in the OPEN group than in the MIS group (*P* = .013). Blood loss was significantly less in the MIS group than in the OPEN group (*P* < .001). The morbidity rate was lower in the MIS group than in the OPEN group, but the difference was not significant (28% and 48%, respectively, *P* = .123). The rate of major morbidities was comparable between the OPEN and MIS groups (8% and 4%, respectively, *P* = .597), and there was no mortality in either group. The duration of postoperative hospitalization tended to be shorter in the MIS group than in the OPEN group (*P* = .073).

**Table 2:** Comparison of short-term outcomes between OPEN and MIS.

Short-term outcomes (n = 75)		OPEN (n = 25), n (%)	MIS (n = 50), n (%)	*P* value
Surgical data				
Surgical procedure	Right-sided colectomy	3 (12%)	13 (26%)	.268
	Left-sided colectomy	5 (20%)	7 (14%)	
	Rectal resection	16 (64%)	30 (60%)	
	Total colectomy	1 (4%)	0 (0%)	
Multivisceral resection	Total	17 (68%)	25 (50%)	.217
Details of resected organs	Abdominal wall muscle	0 (0%)	2 (4%)	.216
	Abdominal wall muscle, superior vesicall artery	0 (0%)	1 (2%)	
	Bladder	0 (0%)	3 (6%)	
	Bladder, prostate	6 (24%)	2 (4%)	
	Bladder, prostate, colon	1 (4%)	0 (0%)	
	Bladder, uterus, ovary	2 (8%)	0 (0%)	
	Colon (the other site apart from primary tumor)	0 (0%)	2 (4%)	
	Gonadal vessels	0 (0%)	1 (2%)	
	Levator ani muscle	1 (4%)	1 (2%)	
	Levator ani muscle, vagina	0 (0%)	1 (2%)	
	Omentum	0 (0%)	1 (2%)	
	Perineal skin	0 (0%)	1 (2%)	
	Peritoneum	0 (0%)	2 (4%)	
	Small intestine	3 (12%)	2 (4%)	
	Uterus	0 (0%)	1 (2%)	
	Uterus, ovary	3 (12%)	4 (8%)	
	Vagina	1 (4%)	1 (2%)	
Lymph node harvest*		19 (0–52)	20 (3–56)	.206
Surgical time (min)*		322 (104–588)	203 (108–787)	.013
Blood loss (mL)*		560 (0–2480)	0 (0–1845)	<.001
Postoperative course				
Morbidity (Clavien-Dindo *>* I)	Total	12 (48%)	14 (28%)	.123
Major morbidity (Clavien-dindo *>* III)	Total	2 (8%)	2 (4%)	.597
	Anastomotic leakage	1 (4%)	0 (0%)	
	Intraabdominal abscess	1 (4%)	0 (0%)	
	Wound dehiscence	0 (0%)	1 (2%)	
	Ureter stenosis	0 (0%)	1 (2%)	
Mortality		0 (0%)	0 (0%)	1.000
Hospitalization (d)*		19 (8–71)	14 (7–65)	.073

**Figure 2.**
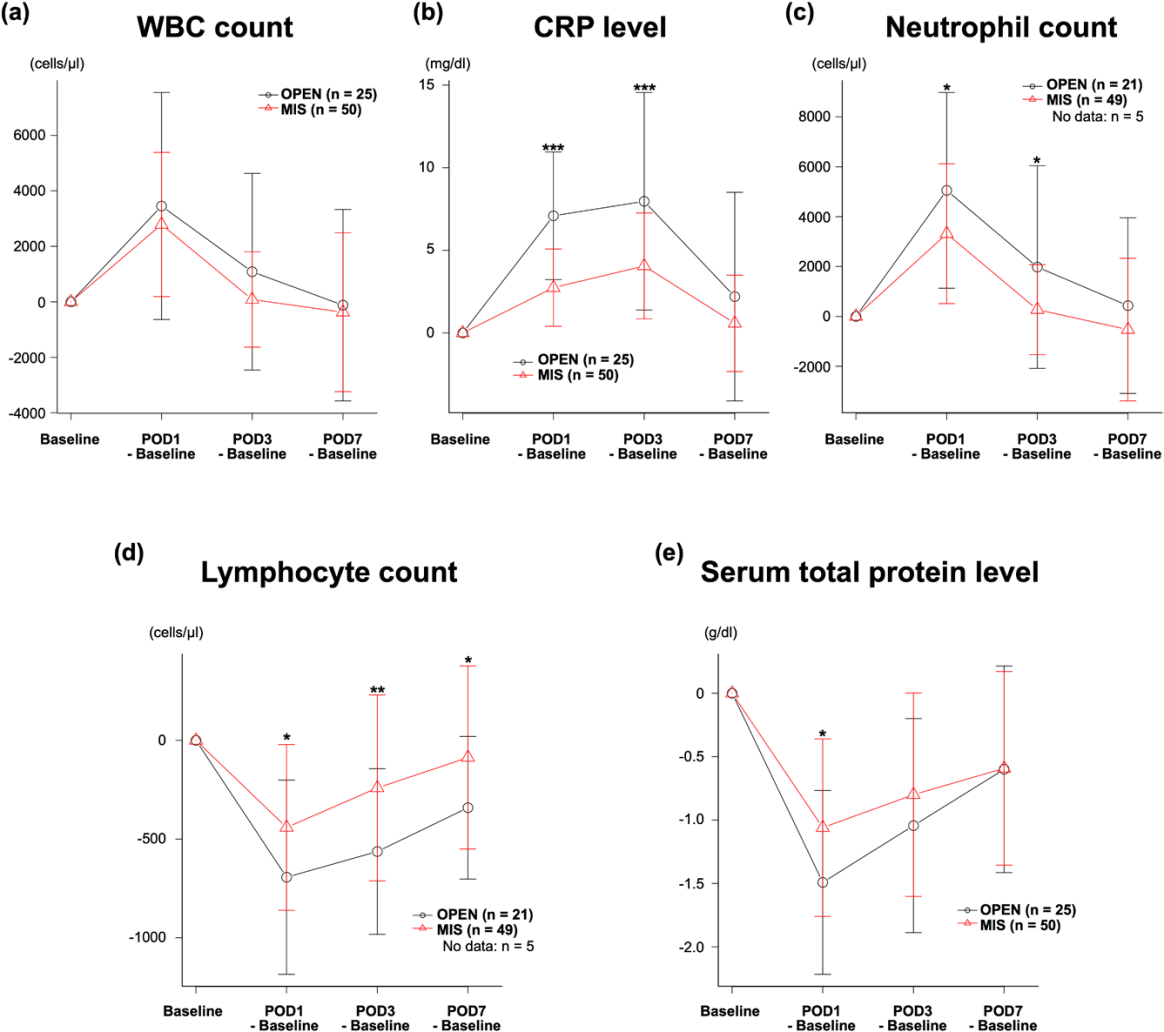
Perioperative alterations in laboratory data using the numeral difference between the postoperative and preoperative data. (A) WBC count. (B) CRP level. (C) Neutrophil count. (D) Lymphocyte count. (E) Serum total protein level. *P < .05, **P < .01, ***P < .001. Black circles and red triangles represent the mean values of the OPEN and MIS groups, respectively. Error bars represent the standard division. Postoperative data were calculated by using the numeral difference between the postoperative and preoperative data. Preoperative data were indicated as the baseline. *P*-values were calculated by using the 2-tailed Student *t* test. CRP = C-reactive protein, MIS = minimally invasive surgery, OPEN = open surgery, POD = postoperative day, WBC = white blood cell.

Perioperative alterations in the laboratory data were calculated by using the numeral difference between postoperative and preoperative data (baseline), and these findings are shown in Figure 2A to E. The neutrophil count on POD 1 (*P* = .040) and 3 (*P* = .017), and the CRP level on POD 1 and 3 (both *P* < .001) were significantly lower in the MIS group than in the OPEN group. In contrast, the lymphocyte counts on POD 1 (*P* = .032), 3 (*P* = .009), and 7 (*P* = .029) and the TP level on POD 1 (*P* = .015) were significantly higher in the MIS group than in the OPEN group.

### 3.2. Pathological findings and postoperative chemotherapy

The pathological findings are shown in Table 3. A total of 51 patients (68%) had pathological T4 tumors. The tumor size, histological grade, and pathological T factor were similar between the OPEN and MIS groups. The pathological N factor was significantly more advanced in the MIS group than in the OPEN group (*P* < .001). pStage II tumors were more commonly observed in the OPEN group than in the MIS group (48% vs 18%), while pStage III tumors were more common in the MIS group than in the OPEN group (12% vs 40%). The number of patients with pStage IV tumors were comparable between the 2 groups (40% vs 42%). The number of patients with positive resection margins was lower in the MIS group than in the OPEN group, but the difference was not significant (12% vs 4%, *P* = .326).

**Table 3:** Comparison of pathological findings between OPEN and MIS.

Pathological findings (n = 75)		OPEN (n = 25), n (%)	MIS (n = 50), n (%)	*P* value
Tumor size (mm)*		70 (31–110)	55 (22–115)	.131
Histological grade	High grade	2 (8%)	10 (20%)	.316
	Low grade	23 (92%)	40 (80%)	
Pathological T factor	T3	6 (24%)	18 (36%)	.460
	T4a	8 (32%)	17 (34%)	
	T4b	11 (44%)	15 (30%)	
Pathological N factor	N0	16 (64%)	13 (26%)	<.001
	N1	7 (28%)	12 (24%)	
	N2	2 (8%)	25 (50%)	
Pathological stage	II	12 (48%)	9 (18%)	.009
	III	3 (12%)	20 (40%)	
	IV	10 (40%)	21 (42%)	
Resection margin positive of primary tumor		3 (12%)	2 (4%)	.326

**Figure 3.**
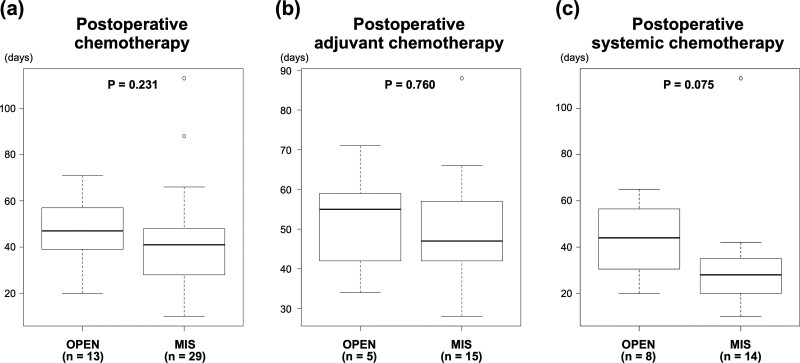
Interval length between surgery and postoperative chemotherapy. (A) Patients treated with postoperative chemotherapy (n = 42). (B) Patients treated with postoperative adjuvant chemotherapy (n = 20). (C) Patients treated with postoperative systemic chemotherapy (n = 22). Box plots represent median values and interquartile ranges. Boxplot whiskers indicate the 10th and 90th percentile values. *P*-values were calculated by using the nonparametric Mann–Whitney *U* test.MIS = minimally invasive surgery, OPEN = open surgery.

Forty-nine patients underwent postoperative chemotherapy. Of these, 7 patients were excluded because chemotherapy was delayed in 5 patients owing to the treatment of metastatic lesions or other diseases after primary tumor resection, and the chemotherapy schedule in 2 patients was unknown as they underwent treatment in different hospitals. Finally, 42 patients were included in this analysis. There was no significant difference in the interval from chemotherapy to surgery between the OPEN and MIS groups (Fig. 3A). Moreover, the time when early postoperative chemotherapy should be initiated depends on the purpose of chemotherapy. Hence, the 42 patients were divided into 2 groups of 20 patients treated with adjuvant chemotherapy and 22 patients treated with systemic chemotherapy. The interval from adjuvant chemotherapy to surgery was similar between the OPEN and MIS groups (Fig. 3B). However, systemic chemotherapy tended to be started earlier, although not significantly, in the MIS group than in the OPEN group (median, 28 and 44 days, respectively, *P* = .075; Fig. 3C).

### 3.3. Long-term oncologic outcomes and prognostic factors for OS and RFS

**Figure 4.**
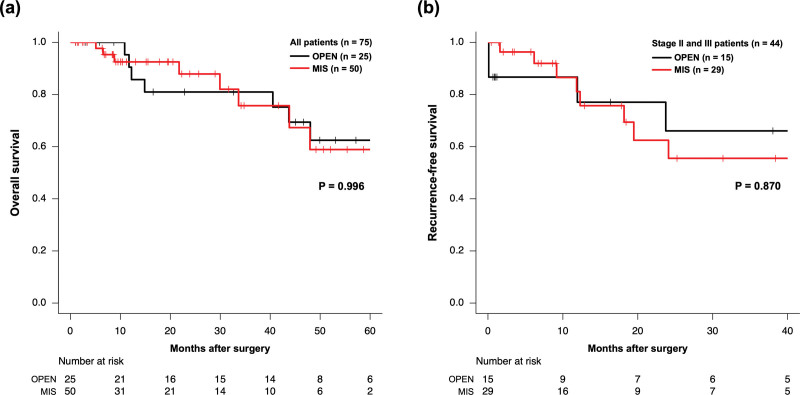
Kaplan–Meier curves for overall survival and recurrence-free survival. (A) All patients (n = 75) for overall survival. (B) Stage II and III patients (n = 44) for recurrence-free survival.MIS = minimally invasive surgery, OPEN = open surgery. *P*-values were calculated by using the log-rank test.

The median follow-up was 19.7 months (range, 1.0–87.1 months). For 75 patients, the 3-year OS rate was 75.7% and 81.0% for the MIS and OPEN groups, respectively (Fig. 4A). For 44 patients with stage II and III cancers, the 3-year RFS was 47.0% and 60.3% for the MIS and OPEN groups, respectively (Fig. 4B). With regard to recurrence, 3 cases had local recurrence and 1 case had distant metastasis in the OPEN group; no case had local recurrence, and 8 cases hade distant metastasis, in the MIS group. There was a significant difference in the local recurrence rate (*P* = .034) between the MIS and OPEN groups. On multivariate analyses, age, sex, tumor location, preoperative treatment, surgical approach, histological grade, pathological T factor, pathological N factor, and distant metastasis (only in OS) were included as covariate factors. Although preoperative treatment and distant metastasis were independent significant prognostic factors for OS, MIS was not a significant factor for OS and RFS (Table 4).

**Table 4:** Prognostic factors for overall survival and recurrence-free survival in multivariate analyses.

Prognostic factors		Overall survival (n = 75)			Recurrence-free survival (n = 44)		
		n	HR (95% CI)	*P* value	n	HR (95% CI)	*P* value
Age	≥70 y	26	1.111 (0.272–4.537)	.884	17	3.500 (0.822–14.90)	.090
	<70 y	49	Reference		27	Reference	
Sex	Male	40	1.476 (0.427–5.096)	.538	29	1.064 (0.263–4.296)	.931
	Female	35	Reference		15	Reference	
Tumor location	Right	18	2.637 (0.848–8.205)	.094	8	0.174 (0.019–1.555)	.118
	Left/rectum	57	Reference		36	Reference	
Preoperative treatment	Yes	21	3.862 (1.055–14.14)	.041	9	1.243 (0.275–5.622)	.778
	No	54	Reference		35	Reference	
Surgical approach	MIS	50	1.036 (0.315–3.409)	.954	29	0.760 (0.155–3.714)	.734
	OPEN	25	Reference		15	Reference	
Histological grade	High grade	12	2.177 (0.574–8.261)	.253	7	3.572 (0.524–24.35)	.194
	Low grade	63	Reference		37	Reference	
Pathological T factor	T4	51	0.972 (0.220–4.298)	.970	27	2.489 (0.451–13.74)	.296
	T3	24	Reference		17	Reference	
Pathological N factor	*>*N1	46	1.934 (0.593–6.305)	.274	22	3.876 (0.862–17.43)	.077
	N0	29	Reference		22	Reference	
Distant metastasis	Yes	31	6.995 (1.720–28.45)	.007			
	No	44	Reference				

## 4. Discussion

In the current study, our results indicated that MIS has a safe postoperative course and tends to have shorter hospitalization duration after surgery than OPEN does, although we recognize that cases requiring MVR with reconstruction were more frequently managed with OPEN than with MIS. The perioperative alteration in laboratory data may reveal that MIS is less invasive and results in enhanced recovery from surgery. Accordingly, postoperative chemotherapy might be initiated earlier for patients with pStage IV tumors who had undergone primary tumor resection via MIS than those who had undergone OPEN, thereby suggesting the advantage of MIS for T4 CRC over OPEN. The long-term oncologic outcomes were comparable between MIS and OPEN.

MIS for T4 CRC has been introduced systematically in our department, as described in the Materials and Methods section. Although urinary system reconstruction is difficult to perform completely via laparoscopic surgery,^[26]^ our team included a laparoscopic surgeon from the urology department who cooperated with us and performed this surgery. The current indications for the conventional laparoscopic approach are T4 CRC treated with or without reconstruction. However, the reduced port and robotic approaches have not been performed for patients with CRC treated with reconstruction. Thus, considering the patient characteristics and surgical data in the current study, there were significant differences in the number of previous abdominal surgery and MVR with reconstruction between the MIS and OPEN groups. Laparoscopic surgery for T4 CRC has a high conversion rate and a low R0 resection rate.^[12–20]^ The conversion rate ranges from 3.8% to 24.7%.^[12–21]^ In particular, in cases treated with MVR, the conversion rate ranged from 6.7% to 28.9%.^[26–30]^ In the overall cohort of the current study, the conversion rates were 8.8% (10/114) for all cases of T4 CRC and 19.5% (8/41) for cases treated with MVR. Thus, MIS, which is technically challenging, is a feasible procedure for treating T4 CRC if it is introduced systematically.

Laparoscopic surgery for CRC improved the short-term outcomes better than OPEN.^[1–5,8]^ However, T4 CRC was excluded in many studies. Moreover, the results of some retrospective studies showed no differences in morbidity and mortality between laparoscopic procedures and OPEN for T4 CRC.^[12,15–17]^ The morbidity rates ranged from 7.7% to 33%.^[12–17]^ In the present study, the rates of major morbidities rates were not different between MIS and OPEN. Furthermore, there was no mortality in both the MIS and OPEN groups. In fact, MIS tended to result in shorter postoperative hospitalization than OPEN. The rate of a positive resection margin of the primary tumor was lower in the MIS group than in the OPEN group. We consider that these results show the safety and potential improvement of the surgical outcome by MIS.

Perioperative alterations in laboratory data indicated less invasiveness and enhanced recovery from surgery in the MIS group compared to the OPEN group. In particular, MIS suppressed the increase in the white blood cell and neutrophil counts and the CRP level. These alterations indicated the less invasiveness of MIS compared with OPEN. Moreover, MIS preserved a higher count of lymphocytes and higher level of TP as compared to OPEN. These alterations revealed that the immune response was preserved and that protein catabolism was suppressed after surgery. Accordingly, the results of the current study may indicate that MIS promoted early recovery after surgery.

Patients with clinical T4 CRC will probably need to receive postoperative chemotherapy. In the current study, pathological T4 CRC was observed in 68% of the patients and metastatic CRC in 41% of patients. Patients without lymph node and distant metastases will probably be classified as having stage II high-risk disease and show the indications for postoperative adjuvant chemotherapy. Patients with distant metastasis require systemic chemotherapy after primary tumor resection for the control of metastasis. The initiation of adjuvant chemotherapy is recommended within 8 weeks after surgery.^[31,32]^ Hence, adjuvant chemotherapy does not need to be initiated very soon. However, systemic chemotherapy should be initiated as early as possible for patients with distant metastasis. In the present study, there was no difference in the interval from surgery to the initiation of adjuvant chemotherapy between MIS and OPEN. In contrast, systemic chemotherapy after primary tumor resection for patients with distant metastasis was not a significant factor, but it tended to be initiated earlier after MIS than after OPEN. This suggests that the suppression of inflammation and the preservation of immune response owing to surgery, as mentioned above, promote recovery and enable the early transition to the subsequent treatment. In a previous study, there was no difference in the interval from surgery to adjuvant chemotherapy between patients treated with laparoscopic surgery and those treated with OPEN (34 and 36 days, respectively).^[16]^ However, no study has evaluated the interval between surgery and postoperative chemotherapy in patients with distant metastasis.

The long-term oncological outcomes are comparable between open and laparoscopic surgeries for CRC, as shown in large randomized-controlled trials; however, T4 CRC cases were excluded in many studies.^[1,2,6,7,9]^ According to the findings of the JCOG 0404 trial, patients with cT4 CRC who underwent laparoscopic surgery tended to show a worse survival compared to those treated with OPEN.^[9]^ However, some retrospective studies have shown no differences in the long-term outcomes between these surgeries.^[12,15–19]^ Although there was a pathological difference in that more advanced nodal status tumors have been included in the MIS group than in the OPEN group, the results of the current study also showed no difference between MIS and OPEN for T4 CRC. However, no cases with local recurrence were observed in the MIS group. Therefore, the MIS group potentially has longer RFS and OS than the OPEN group. Moreover, for patients with pStage IV tumors, subsequent chemotherapy could be initiated early after MIS, thereby possible improving the prognosis.^[26]^

The current study has some limitations. First, as this study was a retrospective review of data from a single center, a large prospective study will be required to verify the new findings. Second, as mentioned in the Materials and methods section, cases treated with conversion surgery were excluded in this study, because we aimed to clarify the advantages of MIS. Third, this study had selection bias because of its retrospective design. Especially, the clinical T4 subcategory and preoperative treatments showed significant differences between OPEN and MIS before matching. Thus, a matched-pair analysis was performed for the removal of the bias as much as possible. Finally, the follow-up period was relatively short for discussing the long-term results. The present results should be examined in the future.

In conclusion, MIS was surgically safe and showed similar oncological outcomes to OPEN. Although selection bias was involved, this study suggests that MIS would have the advantages of reduced invasiveness and enhanced recovery from surgery. Therefore, patients undergoing MIS might receive subsequent postoperative treatments earlier. These findings can improve the prognosis of patients with T4 CRC.

### Author contributions

KI, SH, YM, HM, NI, TY, NT, and AT conceptualized and designed the study. KI and SH wrote the manuscript. KI performed the statistical analyses. KI, SH, YM, HM, NI, TY, and NT performed the operations and collected clinicopathological data. AT supervised the study. KI, SH, YM, HM, NI, TY, NT, and AT interpreted the results and participated in writing the report. All authors read and approved the final manuscript.

### Acknowledgments

The authors thank Isao Yokota for advice on statistical analysis.

## Footnotes

* Median (range).

† There is some duplication.

* Median (range).

* Median (range).
